# Polymorphisms in CARD8 and NLRP3 are associated with extrapulmonary TB and poor clinical outcome in active TB in Ethiopia

**DOI:** 10.1038/s41598-019-40121-8

**Published:** 2019-02-28

**Authors:** Ebba Abate, Robert Blomgran, Deepti Verma, Maria Lerm, Mats Fredrikson, Meseret Belayneh, Peter Söderkvist, Olle Stendahl, Thomas Schön

**Affiliations:** 1 Division of Microbiology Infection and Inflammation, Department of Clinical and Experimental Medicine, Linköping University, Linköping, Sweden; 2 Ethiopian Public Health Institute, EPHI, Addis Abeba, Ethiopia; 3 Department of Clinical and Experimental Medicine, Division of Rheumatology, Linköping University, Linköping, Sweden; 4 Department of Clinical and Experimental Medicine, Division of Environmental and Occupational Medicine, Faculty of Medicine Linköping University, Linköping, Sweden; 5 Department of Medical Laboratory Sciences, University of Addis Abeba, Addis Abeba, Ethiopia; 6 Division of Cell Biology, Department of Clinical and Experimental Medicine, Linköping University, Linköping, Sweden; 7 Department of Infectious Diseases and Clinical Microbiology, Kalmar County Hospital, Linköping University, Linköping, Sweden

## Abstract

Innate immunity is a first line defense against *Mycobacterium tuberculosis* infection where inflammasome activation and secretion of the pro-inflammatory cytokine IL-1beta, plays a major role. Thus, genetic polymorphisms in innate immunity-related genes such as CARD8 and NLRP3 may contribute to the understanding of why most exposed individuals do not develop infection. Our aim was to investigate the association between polymorphisms in CARD8 and NLRP3 and active tuberculosis (TB) as well as their relationship to treatment outcome in a high-endemic setting for TB. Polymorphisms in CARD8 (C10X) and NLRP3 (Q705K) were analysed in 1190 TB patients and 1990 healthy donors (HD). There was a significant association between homozygotes in the CARD8 polymorphism and extrapulmonary TB (EPTB), which was not the case for pulmonary TB or HDs. Among TB-patients, there was an association between poor treatment outcome and the NLRP3 (Q705K) polymorphism. Our study shows that inflammasome polymorphisms are associated with EPTB and poor clinical outcome in active TB in Ethiopia. The practical implications and determining causal relationships on a mechanistic level needs further study.

## Introduction

One peculiar feature of tuberculosis (TB) is that relatively few individuals become infected even after long-term household exposure^1,2^. It has been estimated that only 20–50% of exposed household contacts develop latent TB within 2 years^2^. Thus, the remaining 50–80% mount an effective innate immune response which clears the bacteria even before an adaptive cell-mediated immune response develops^3^. Innate immunity reactions include mechanisms such as Toll-like receptor signaling and inflammasome activation leading to autophagy, release of pro-inflammatory cytokines, nitric oxide, and vitamin D-mediated antimicrobial peptide production^3,4^.

Inflammasome activation is critical for the clearance of bacterial pathogens and removal of damaged cells, but uncontrolled inflammasome activation can also enhance autoimmune disorders^3–5^. In Crohn’s disease and rheumatoid arthritis (RA), a hyperactive inflammasome contributes to an excessive inflammatory response^6,7^. In these cases, single nucleotide polymorphisms (SNPs) leading to a constitutively active NLRP3 inflammasome contribute to the disease manifestations. NLRP3 is a cytosolic pattern-recognition receptor that in response to pathogens and danger molecules mediates production of pro-inflammatory cytokines^8^. NLRP3 activation leads to the recruitment of the adaptor ASC,which binds to NLRP3, and through its caspase recruitment domain (CARD) binds procaspase-1. Caspase-1 is subsequently activated to a catalytically active protease, which leads to the secretion of IL1-beta^8^. The Q705K polymorphism in NLRP3 has shown to be a gain-of-function SNP leading to enhanced IL1-beta release^5^.

Caspase recruitment domain-containing protein 8 (CARD8), was recently shown to be a specific suppressor of NLRP3/ASC/procaspase −1 protein assembly, thereby inhibiting NLRP3-inflammasome activation^9^. The C10X polymorphism in CARD8 leads to a truncated, non-functional protein with loss in CARD8-mediated inhibition of caspase-1.

Gain-of-function polymorphisms in the NLRP3 inflammasome resulting from gene polymorphisms such as NLRP3 (Q705K) and loss-of-function in relation to CARD8 (C10X) are associated to RA and Crohn’s disease, and some evidence for a combined effect of these polymorphisms have been described^6,7^.

*M*. *tuberculosis*, which replicates inside macrophages, uses its Esx-1 secretion machinery and the membrane-damaging protein ESAT-6 to access the cytoplasmic compartment, thereby activating the NLRP3 inflammasome^10^. Furthermore, macrophages from individuals with combined CARD8 (C10X) and NLRP3 (Q705K) polymorphisms have been shown to limit intracellular growth of *M*. *tuberculosis* H37Rv more efficiently compared to wild-type cells^11^, suggesting a selective advantage for carriers of these polymorphisms in an TB endemic setting.

With this background, the association between SNPs in the NLRP3 inflammasome (Q705K) or its regulatory protein CARD8 (C10X) and active TB including treatment response needs further investigation in large epidemiological studies in high-endemic areas. Our aim was therefore to explore the association between established polymorphisms (NLRP3 (Q705K) and CARD8 (C10X)) and pulmonary TB (PTB) as well as extrapulmonary TB (EPTB) including treatment outcome in Ethiopia, a high endemic setting for TB and in particular EPTB.

## Results

### Genotype distribution and allele frequencies in TB patients and healthy controls

In total, 1190 HIV negative TB patients and 1990 controls (HDs) were included. SNPs were found to be in Hardy–Weinberg equilibrium (p = 0.79 and 0.99 for NLRP3 and CARD8 respectively). The median age in TB patients and HDs were 30 (IQR: 23–45) years and 25 (22–32) years, respectively. Female to male ratio in TB patients and HDs was 552/638 and 440/1546, respectively.

The overall genotype and allele distribution of NLRP3 (Q705K) and CARD8 (C10X) is shown in Table 1. Association analyses for the co-dominant, dominant and recessive model are presented in Table 2. Overall, homozygotes in NLRP3 (Q705K) were very rare, and present in 0.1% and 0.25% of the HDs and TB patients, respectively. The recessive model showed a significantly higher presence of homozygotes in CARD8 (C10X) in EPTB (19.9% 69/347) compared to pulmonary TB (PTB) (odds ratio (OR): 1.8 (1.3–2.6); 12.0%; 91/759, p < 0.001) or HDs (OR: 1.4 (1.05–1.9); 15.1%; 296/1964, p = 0.024; Table 3). When adjusted for age and sex, the comparison between PTB and EPTB remained significant (adjusted odds ratio (aOR): 1.8 (1.3–2.5) p < 0.001). Females who were homozygotes in CARD8 (C10X) showed an inverse association to PTB (Table 4). An analysis of the combined effects of all genotype combinations of CARD8 (C10X) and NLRP3 (Q705K) adjusted for age and sex, showed no significant effect on the association to TB nor PTB or EPTB separately (p = 0.46, 0.39 and 0.83 respectively for homozygotes in CARD8 (TT) and heterozygotes in NLRP3 (CA)). No statistically significant associations between CARD8 (C10X) and active TB including PTB or EPTB were observed in the co-dominant or the dominant model (Tables 2 and 3).

**Table 1:** Genotype and allele distribution of NLRP3 (Q705K) and CARD8 (C10X) in healthy donors (HD) and all patients with active tuberculosis (TB).

Locus	Genotype/allele	All TB, n (%)	HD, n (%)	OR (95% CI)	p
NLRP3 (Q705K)	CC	1076 (90.65)	1791 (90.8)	0.97 (0.76–1.3)	0.871
rs35829419	CA	108 (9.10)	179 (9.1)	1.0 (0.78–1.3)	0.984
	AA	3 (0.25)	2 (0.10)	2.50 (0.42–15.0)	0.317
	C	1184 (91.4)	1970 (91.6)	0.98 (0.77–1.3)	0.873
	A	111 (8.6)	181 (8.4)	1.0 (0.80–1.3)	0.873
CARD8 (C10X)	AA	412 (37.08)	734 (37.37)	0.99 (0.85–1.2)	0.874
rs2043211	AT	539 (48.51)	934 (47.56)	1.0 (0.90–1.2)	0.609
	TT	160 (14.40)	296 (15.07)	0.95 (0.77–1.2)	0.616
	A	951 (57.6)	1668 (57.6)	1.0 (0.89–1.1)	0.958
	T	699 (42.4)	1230 (42.4)	0.99 (0.88–1.1)	0.958

**Table 2:** Association of NLRP3 (Q705K) and CARD8 (C10X) to all TB patients compared to healthy donors in co-dominant, dominant and recessive models.

Locus	Model	Genotype	All TB, n (%)	HD, n (%)	OR (95% CI)	p
NLRP3 (Q705K)	Codominant	CC	1076 (90.65)	1791 (90.8)	1	
rs35829419		CA	108 (9.10)	179 (9.1)	0.96 (0.72–1.3)	0.760
		AA	3 (0.25)	2 (0.10)	3.6 (0.60–21.7)	0.160
	Dominant	CC	1076 (90.65)	1791 (90.8)	1	
		CA + AA	111 (9.35)	181 (9.2)	0.97 (0.76–1.3)	0.871
	Recessive	AA	3 (0.25)	2 (0.10)	1	
		CC + CA	1184 (99.75)	1970 (99.9)	2.50 (0.42–15.0)	0.317
CARD8 (C10X)	Codominant	AA	412 (37.08)	734 (37.37)	1	
rs2043211		AT	539 (48.51)	934 (47.56)	1.0 (0.88–1.2)	0.735
		TT	160 (14.40)	296 (15.07)	0.96 (0.77–1.2)	0.745
	Dominant	AA	412 (37.0)	734 (37.4)	1	
		AT + TT	699 (63.0)	1229 (62.6)	0.99 (0.85–1.2)	0.874
	Recessive	TT	160 (14.4)	296 (15.1)	1	
		AA + AT	951 (86.6)	1668 (84.9)	0.95 (0.77–1.2)	0.616

**Table 3:** Association of NLRP3 (Q705K) and CARD8 (C10X) to EPTB patients compared to healthy donors in co-dominant, dominant and recessive models.

Locus	Model	Genotype	EPTB, n (%)	HD, n (%)	OR (95% CI)	p
NLRP3 (Q705K)	Codominant	CC	329 (89.9)	1791 (90.8)	1	
rs35829419		CA	37 (10.1)	179 (9.1)	1.1 (0.77–1.6)	0.578
		AA	0 (0)	2 (0.10)	—	—
	Dominant	CC	329 (89.9)	1791 (90.8)	1	
		CA + AA	37 (10.1)	181 (9.2)	0.90 (0.62–1.3)	0.574
	Recessive	AA	0 (0)	2 (0.10)	1	
		CC + CA	366 (100)	1970 (99.9)	1.1 (0.052–22.4)	0.963
CARD8 (C10X)	Codominant	AA	123 (35.5)	734 (37.37)	1	
rs2043211		AT	155 (44.7)	934 (47.56)	1.0 (0.78–1.3)	0.953
		TT	69 (19.9)	296 (15.07)	1.4 (1.0–1.9)	0.051
	Dominant	AA	123 (35.5)	734 (37.4)	1	
		AT + TT	224 (64.5)	1229 (62.6)	0.92 (0.72–1.2)	0.489
	Recessive	TT	69 (19.9)	296 (15.1)	1	
		AA + AT	278 (81.1)	1668 (84.9)	1.4 (1.05–1.9)	0.024

**Table 4:** Association of NLRP3 (Q705K) and CARD8 (C10X) to all TB patients, EPTB and PTB patients compared to healthy donors in a co-dominant model of females only, adjusted for age.

Locus	Genotype	All TB Cases	*p*	PTB	*p*	EPTB	*p*
		OR (95% CI)		OR (95% CI)		OR (95% CI)	
NLRP3 (Q705K)	CC	1		1		1	
rs35829419	CA	1.13 (0.68–1.86)	0.645	1.30 (0.76–2.22)	0.332	0.66 (0.30–1.43)	0.295
	AA	0.65 (0.032–12.7)	0.774	0.94 (0.05–18.69)	0.967	—	—
CARD8 (C10X)	AA	1		1		1	
rs2043211	AT	0.89 (0.66–1.22)	0.477	0.87 (0.62–1.22)	0.418	0.87 (0.56–1.37)	0.573
	TT	0.91 (0.59–1.41)	0.683	0.57 (0.34–0.98)	0.040	1.66 (0.95–2.90)	0.073

### Association between polymorphisms in CARD8 and NLRP3, and clinical outcome during treatment of active tuberculosis

Overall, males had an increased frequency of poor treatment outcome compared to females (6.8% (25/370) vs 1.6% (6/377), p < 0.001). The overall genotype and allele distribution of NLRP3 (Q705K) and CARD8 (C10X) in relation to treatment outcome is shown in Table 5 and the corresponding association analyses for the co-dominant, dominant and recessive model are presented in Table 6. Within the TB group, there was a strong association to poor treatment outcome in those heterozygous for Q705K of NLRP3 (aOR: 3.09 (1.09–8.80), p = 0.035, Table 7) in a co-dominant model adjusted for age and sex. This association was particularly strong for females (aOR: 10.66 (2.06–55.26), p = 0.005). An analysis of the combined effects of all genotype combinations NLRP3 (Q705K) and CARD8 (C10X) adjusted for age and sex showed a possible association between heterozygotes in NLRP3 (Q705K) and homozygotes in CARD8 (C10X) (aOR 8.3 (0.76–90.3), p = 0.083) and risk for poor treatment outcome.

**Table 5:** Genotype and allele distribution of NLRP3 (Q705K) and CARD8 (C10X) in relation to treatment outcome in all patients with active tuberculosis (TB).

Locus	Genotype/allele	Poor outcome, n (%)	Successful outcome, n (%)	OR (95% CI)	p
NLRP3 (Q705K)	CC	16 (76.2)	602 (90.7)	0.32 (0.11–0.93)	0.036
rs35829419	CA	5 (23.8)	61 (9.2)	3.1 (1.1–8.7)	0.033
	AA	0 (100)	1 (0.1)	—	—
	C	21 (80.8)	663 (91.4)	0.39 (0.14–1.1)	0.070
	A	5 (19.2)	62 (8.6)	2.5 (0.92–7.0)	0.070
CARD8 (C10X)	AA	6 (35.3)	227 (37.2)	0.92 (0.34–2.5)	0.872
rs2043211	AT	8 (47.1)	292 (47.9)	0.97 (0.37–2.5)	0.947
	TT	3 (17.6)	91 (14.9)	1.2 (0.34–4.3)	0.756
	A	14 (56.0)	519 (57.5)	0.93 (0.42–2.1)	0.878
	T	11 (44.0)	383 (42.5)	1.1 (0.50–2.4)	0.878

**Table 6:** Association between NLRP3 (Q705K) and CARD8 (C10X) genotypes and poor treatment outcome in all TB patients in co-dominant, dominant and recessive models.

Locus	Model	Genotype	Poor outcome, n (%)	Successful outcome, n (%)	OR (95%CI)	p
NLRP3	Codominant	CC	16 (76.2)	602 (90.7)	1	
(Q705K)		CA	5 (23.8)	61 (9.2)	3.09 (1.1–8.8)	0.035
rs35829419		AA	0 (100)	1 (0.1)	—	—
	Dominant	CC	16 (76.2)	602 (90.7)	1	
		CA + AA	5 (23.8)	62 (9.3)	0.32 (0.11–0.93)	0.036
	Recessive	AA	0 (0)	1 (0.2)	—	—
		CC + CA	21 (100)	663 (99.8)	—	—
CARD8	Codominant	AA	6 (35.3)	227 (37.2)	1	
(C10X)		AT	8 (47.1)	292 (47.9)	1.0 (0.35–3.0)	0.957
rs2043211		TT	3 (17.6)	91 (14.9)	1.2 (0.30–5.0)	0.781
	Dominant	AA	6 (35.3)	227 (34.4)	1	
		AT + TT	11 (64.7)	383 (62.8)	0.92 (0.34–2.5)	0.872
	Recessive	TT	3 (17.6)	91 (14.9)	1	
		AA + AT	14 (82.4)	519 (85.1)	1.2 (0.34–4.3)	0.756

**Table 7:** Association between NLRP3 (Q705K) and CARD8 (C10X) genotypes and poor treatment outcome in all TB patients, EPTB and PTB patients respectively in a co-dominant, model.

Gene (Marker)	Genotype	All Cases	*p*	PTB	*p*	EPTB	*p*
		OR (95% CI)		OR (95% CI)		OR (95% CI)	
NLRP3 (Q705K)	NLRP3						
rs35829419	CA	3.09 (1.09–8.80)	0.035	3.92 (1.33–11.53)	0.013	—	—
	AA	—	—	—	—	—	—
CARD8 (C10X)	CARD8						
rs2043211	AT	1.03 (0.35–3.02)	0.957	1.10 (0.34–3.56)	0.867	0.62 (0.37–10.48)	0.195
	TT	1.22 (0.30–5.03)	0.781	1.70 (0.39–7.40)	0.481	—	—

## Discussion

The main result of this large-scale study on inflammasome polymorphisms in Ethiopia is an association between homozygotes in CARD8 (C10X) and EPTB as well as an association to an impaired TB treatment outcome in carriers of the NLRP3 (Q705K) polymorphism.

The distribution of homozygotes for the CARD8 (C10X) polymorphism is much less frequent in the European population (9.9%; 99/1003) compared to Ethiopian blood donors in the current study (15.1%, p < 0.001)^12^. The rate of homozygotes (19.9%) in the CARD8 (C10X) polymorphism in EPTB patients is twice as high as in European blood donors (9.9%). We found a significantly higher frequency in CARD8 (C10X) homozygotes in EPTB patients from Gondar, Ethiopia in relation to healthy blood donors from the same area, which indicates that CARD8 (C10X) may be one contributing factor to the unexplained high rate of EPTB in this region^13^.

In a previous study, macrophages from heterozygotes in CARD8 (C10X) had a significantly impaired capacity to restrict *M*. *tuberculosis* growth. On the other hand, macrophages isolated from three combined heterozygote carriers of both polymorphisms (NLRP3 (Q705K)/CARD8 (C10X)) displayed enhanced anti-mycobacterial activity and increased IL1-beta production^11^. The interaction between the inflammasome and *M*. *tuberculosis* should be further investigated as our current data combined with these pre-clinical observations indicate a link between loss of control to restrict mycobacterial growth and association to EPTB in carriers of the CARD8 (C10X) polymorphism in the Ethiopian population.

EPTB is commonly manifested as generalized lymphadenopathy with higher rates of inflammation and a stronger pro-inflammatory reaction compared to other clinical forms of TB^4^. In patients with active TB, a hyper-reactive inflammasome may drive the inflammatory state which could prolong the time to cure. Indeed, our data show a significant association between NLRP3 (Q705K) polymorphisms and poor clinical outcome. It is noteworthy that females show an even stronger association between poor clinical outcome and polymorphisms in NLRP3 (Q705K) despite better clinical outcomes in general than males. It has been shown that inflammasome activation may also cause inflammation and tissue damage in patients with TB-associated IRIS, who show early transcriptional responses^14^ including increased levels of IL1-beta and IL18 and enhanced upregulation of NLRP3 mRNA.

To our knowledge, this study is the largest so far to investigate the association between polymorphisms for CARD8 (C10X) and NLRP3 (Q705K) and active TB including treatment outcome. A small study of 288 patients with PTB and healthy controls in Brazil showed an increased risk of PTB in subjects with the rs10754558 polymorphism in NLRP3, but could not confirm an association to active TB in patients with either NLRP3 (Q705K) or CARD8 (C10X) nor in combination^15^. That study did not consider patients with EPTB and there was no report of treatment outcome. Our results show that it is important to analyse different forms of TB separately as it has repeatedly been shown that EPTB is associated to a distinct host response compared to smear positive pulmonary or disseminated TB.

Our study has limitations. The major one is that latent TB screening was not done in blood donors. However, current methods as of tuberculin skin test and interferon-gamma release assays cannot adequately predict the true rate of LTBI, as a positive test is not necessarily associated with the presence of latent bacteria. Furthermore, the diagnosis of EPTB was mainly established clinically but according to WHO criteria and the majority of patients were cured after TB treatment. Although the data indicates an association between inflammasome polymorphisms and EPTB as well as treatment outcome, causal relationships needs to be substantiated by further studies. Despite these limitations, our observations support the hypothesis that inflammasome-driven hyperinflammation may affect the course of TB infection.

In conclusion, we found that polymorphisms in CARD8 (C10X) and NLRP3 (Q705K) leading to a more susceptible inflammasome were associated with EPTB and poor treatment outcome in patients with pulmonary TB, respectively. The results implicate a central role of the inflammasome in TB and may partly explain the high incidence of EPTB in the Ethiopian population.

## Methods

### Ethical considerations

All patients and healthy individuals participated in the study after informed consent only. The study was conducted in accordance with the Declaration of Helsinki and was approved by the ethical review boards of Gondar University (RPO 05/489/2014) and Linköping University (2014/233-31).

### Patients and healthy individuals

Newly diagnosed adult pulmonary TB patients at Gondar University Hospital, Gondar Health Centre and Debark Hospital, Ethiopia were consecutively recruited to the study. WHO-based criteria was used to define PTB and EPTB. HIV positive patients were excluded according to routine investigations by using two different rapid testing kits with confirmation with a third one, if discrepancies occurred. Treatment outcomes for TB patients were recorded according to the latest WHO guidelines. Treatment success included all patients whom were cured or completed treatment, whereas a poor treatment outcome was recorded for those with treatment failure, including death or default. Patients lost to follow up were recorded as uncertain outcome and were not included in the analysis. Apparently healthy blood donors (HDs) were recruited from the same community from the blood bank of the Gondar University Hospital. Active TB was excluded by the routine clinical screening at the blood bank.

### Genotyping by real-time PCR

Heparinized whole blood was collected and stored at −80 °C until DNA extraction in a MagnaPure96 instrument (Roche, USA). Genotyping was performed by a TaqMan® SNP genotyping assay with 7900HT Fast Real-Time PCR system (Applied Biosystems, Foster City, CA, USA) followed by allelic discrimination for the polymorphisms NLRP3 (Q705K, rs35829419) of and CARD8 (C10X, rs2043211). The final reaction contained 2 µL genomic DNA (20 ng), 10 µL 2x TaqMan® Genotyping Master Mix (Applied Biosystems) and 0.5 µL of 20x TaqMan® SNP Genotyping assays. Pre-designed primers and probes and a cut-off of <35 amplification cycles was used.

### Statistical analyses

Descriptive data is presented as medians and interquartile ranges (IQR). Further, chi-square tests were performed to compare genotype including both a dominant (FF vs Ff + ff), codominant (FF vs Ff vs ff) and a recessive (ff vs FF + Ff) model. Binary logistic regression was used to calculate odds ratios (OR), adjusted odds ratios (aOR) and corresponding 95% confidence intervals (95% CI) in the co-dominant model. Interaction between genes in relation to age and sex were adjusted for using logistic regression analysis and p < 0.05 was considered as significant. Hardy–Weinberg equilibrium was calculated in accordance with standard procedures. The results were analysed by Statistica 12.0 software package (Dell Statistica software (version 13, Tulsa, USA)) and STATA 14.1 (StataCorp LLC, College Station, TX, USA).
